# Evidence for the Effectiveness of Feedback from Wearable Inertial Sensors during Work-Related Activities: A Scoping Review

**DOI:** 10.3390/s21196377

**Published:** 2021-09-24

**Authors:** Roger Lee, Carole James, Suzi Edwards, Geoff Skinner, Jodi L. Young, Suzanne J. Snodgrass

**Affiliations:** 1 School of Health Sciences, The University of Newcastle, Newcastle 2308, Australia; Carole.James@newcastle.edu.au (C.J.); Suzanne.Snodgrass@newcastle.edu.au (S.J.S.); 2 Centre for Brain and Mental Health Research, The University of Newcastle, Newcastle 2308, Australia; 3 Centre for Resources Health and Safety, The University of Newcastle, Newcastle 2308, Australia; 4 School of Health Sciences, The University of Sydney, Sydney 2006, Australia; Suzi.Edwards@sydney.edu.au; 5 School of Information and Physical Sciences, The University of Newcastle, Newcastle 2308, Australia; Geoff.Skinner@newcastle.edu.au; 6 Department of Physical Therapy, Bellin College, Green Bay, WI 54311, USA; jodiyoung2@gmail.com

**Keywords:** wearable devices, posture, industrial/workplace ergonomics, feedback

## Abstract

**Background:** Wearable inertial sensor technology (WIST) systems provide feedback, aiming to modify aberrant postures and movements. The literature on the effects of feedback from WIST during work or work-related activities has not been previously summarised. This review examines the effectiveness of feedback on upper body kinematics during work or work-related activities, along with the wearability and a quantification of the kinematics of the related device. **Methods:** The Cinahl, Cochrane, Embase, Medline, Scopus, Sportdiscus and Google Scholar databases were searched, including reports from January 2005 to July 2021. The included studies were summarised descriptively and the evidence was assessed. **Results:** Fourteen included studies demonstrated a ‘limited’ level of evidence supporting posture and/or movement behaviour improvements using WIST feedback, with no improvements in pain. One study assessed wearability and another two investigated comfort. Studies used tri-axial accelerometers or IMU integration (*n* = 5 studies). Visual and/or vibrotactile feedback was mostly used. Most studies had a risk of bias, lacked detail for methodological reproducibility and displayed inconsistent reporting of sensor technology, with validation provided only in one study. Thus, we have proposed a minimum ‘Technology and Design Checklist’ for reporting. **Conclusions:** Our findings suggest that WIST may improve posture, though not pain; however, the quality of the studies limits the strength of this conclusion. Wearability evaluations are needed for the translation of WIST outcomes. Minimum reporting standards for WIST should be followed to ensure methodological reproducibility.

## 1. Introduction

Work-related musculoskeletal disorders (WMSDs) can result from non-traumatic inflammatory or degenerative conditions during work or work-related activities [1]. Dysfunction of muscles, ligaments, tendons, joints and/or cartilage may decrease the overall physiological efficiency within the human body [2]. The most common WMSDs are neck and back pain, which together represent the leading cause of years lived with disability globally [3] and are a debilitating ongoing health concern for many individuals [4,5,6]. Other consequences from WMSDs are economic factors, which may result in lower job satisfaction and psychological wellbeing [7], worker absenteeism, reduced productivity, and increasing business/health-care costs [6,8,9]. Therefore, practical solutions to mitigate and/or manage upper body WMSDs are required.

Poor posture and movement behaviour are likely to contribute to neck, shoulder and/or lower back pain complaints among workers [10,11]. Individuals that engage in awkward upper body postures (non-neutral joint positions) [12,13] and/or poor movement behaviour (e.g., sedentary behaviour) [14,15,16] are likely to sustain a WMSD. Alongside sedentary tasks, manual handling (pushing, pulling, carrying, lifting, holding, moving or restraining an object) [17] and physical exposure [13] represent a large proportion of work-related MSD, as postures are mostly determined by the spatial relationship between the worker and the task. Furthermore, MSDs are multifactorial, for example, increased work stressors, demands and durations of working hours are highly correlated with an increased risk of WRMDs [18], in addition to psychological and behavioural well-being [19,20]. Thus, there is a need to design appropriate workplace interventions to mitigate WMSD risks [21,22] that consider all of these factors.

Designing evidence-based workplace interventions requires a well-designed and rigorous evaluation process [23]. However, results from studies investigating the link between posture and MSD vary [23], with a lack of consensus for current MSD intervention(s) that are likely to be attributed to low patient compliance [24]. Greater rigor in measurement and higher-quality studies are required in order to identify the underlying mechanisms responsible for WMSDs and/or pain development [20,23,25], to improve and further develop current workplace interventions.

Rigorous kinematic evaluation can assist in WMSD management and prevention strategies by improving knowledge of the underlying mechanical, physiological and anatomical factors involved in human motion [26]. Evaluation of the kinematics of workplace activities can be broadly classified into three categories: observational studies, self-reported studies and direct measurements [27]. Although observational assessments and self-reports are widely used [28], their reliance on an observer’s interpretation or an individual’s perception of events may lack objectivity. Direct measurements such as the three-dimensional motion capture system (MOCAP) are the gold standard in kinematic analysis, providing in-depth objective measurements. However, MOCAP systems are expensive, complex and require specialised software [26,29] and are therefore mostly laboratory-based [30], making it difficult to determine functional postures within a real-world working environment [31]. Kinematics measured within an individual’s naturalistic or usual environment is more likely to identify their ‘usual’ or true postures, in comparison to laboratory settings, which typically lack any workplace stressors and/or demands [32].

A recent advancement in wearable technology, incorporating several inertial sensors (an accelerometer, gyroscope and/or a magnetometer) [33], which is able to measure kinematics outside the laboratory, is the inertial measurement unit (IMU). The IMU can detect motion, orientation and heading within a 3D space by performing calculations in terms of acceleration (accelerometer), angular velocity (gyroscope) and rotation (magnetometer), respectively, and can send data wirelessly via Bluetooth or Wi-Fi [34]. An IMU can objectively measure an individual’s body positioning in real-time and within their own environment or workspace [33,35]. Further advancements in customisable software and algorithms provide individualised real-time feedback on posture or movement behaviour [30,36]. Synchronizing multiple IMU devices can operate as a wireless body area network (WBAN) to support detailed biomechanical model development and capture more complex kinematic movement data compared to a single IMU. An IMU has a large range of applications, for example, distinguishing postural differences between individuals [37], or home-based monitoring during rehabilitation to enhance patient compliance and therefore improve functional recovery [38] An inertial sensor is mostly reliable and valid for measuring posture [37,39,40,41]. However, the validity of an inertial sensor is largely dependent on the environment and task performed. Therefore, wherever practical, the inertial sensor(s) should be validated using a gold standard (e.g., a mocap system) prior to their specific usage and environment [30,42]. Validation is paramount for the translation of sensor technology into clinical and rehabilitation settings [42,43,44].

Wearability is described as the interaction between the individual and the sensing equipment [45]. An individual’s task performance may be affected by poor wearability [43]. The consideration of wearability is essential to evaluating the effectiveness of sensor technology, and includes aspects such as sensors’ comfort, mass, appropriate attachment (the prevention of aberrant sensor movement) and obtrusiveness, which may interfere with achieving the user acceptability of the wearable technology [45,46]. Wearability is important for achieving adherence to, and subsequently effectiveness with, workplace interventions using wearable technology [25] and in implementing wearable technology in real-world settings [43].

Wearable inertial sensor technology (WIST) can provide real-time feedback to the wearer. The aim of real-time feedback from WIST is to provide the individual with greater self-awareness of posture and/or movement behaviour during a task and to facilitate changes in order to mitigate or manage musculoskeletal injury. Real-time feedback is a form of extrinsic prompting to assist individuals when intrinsic (internal) feedback mechanisms are weak or compromised, e.g., in cases of stroke or cerebral palsy [43]. Several rehabilitation and clinical studies have reported on the effectiveness of real-time feedback from WIST, for example, in relation to increased range of movement (ROM) [47,48], the retention of motivation during rehabilitation [49] and reduction of lower back pain [48]. Feedback increases self-awareness during functional tasks through goal-directed practice and the repetition of prompts to improve task retention [50], e.g., self-correction of posture through repeated personalised extrinsic prompts. Extrinsic feedback mechanisms are particularly beneficial for patients with stroke, where intrinsic feedback mechanisms are often impaired [49]. Feedback has been reported to improve functional movements and the retention of learning [51,52,53,54]. Given the flexibility of WIST, in terms of its ability to personalise feedback and capture motion in real-time, WIST is becoming more commonly used in movement analysis and neurological rehabilitation settings [43].

Several reviews have researched the use of WIST systems for rehabilitation and motion analysis. A review by Valero, Sivanathan [55] focussing on wearable technology and WMSD within the construction industry reviewed methods to evaluate posture and movement and proposed a new form of WIST to track posture in construction workers. A review by Wang, Markopoulos [43] investigated wearable systems for upper body rehabilitation and found that most were used in studies of patients with stroke. Another review evaluated commercially available WIST devices and evaluated their benefits and limitations [56]. However, no review has summarised the effectiveness of the use of WIST feedback during work or work-related activities to change upper body posture and movement behaviour. Therefore, this scoping review aims to provide a synthesis for the effectiveness of WIST feedback on upper body kinematics during work or work-related activities, as well as the related topics of device wearability and the use of WIST to quantify kinematics. These findings will assist researchers and clinicians by providing knowledge to facilitate the translation of WIST into practice, specifically for upper body work-related activities.

## 2. Materials and Methods

Preliminary literature searches identified limited studies on the effectiveness of WIST feedback; therefore, a scoping review to support a broader set of aims was considered more appropriate than a systematic review, which applies a narrower focus. This review is based upon the modified framework of Daudt, van Mossel [57] for scoping reviews developed by Arksey and O’Malley [58] to assist with the continuum in methodological standards. These authors [57] defined a scoping review as a review mapping literature on a particular topic or research area and reporting upon key aspects, such as research gaps, sources and types of evidence to inform practice. Adhering to the recommendations of these authors [57], this review employed a multidisciplinary team (physiotherapists, occupational therapists, software engineer and biomechanist) to ensure that a diverse range of knowledge and expertise was utilised.

### 2.1. Search Strategies and Search Terms

Six databases were searched from 1 January 2005 to 15 July 2021: Cinahl, Cochrane, Embase, Medline, Scopus, and Sportdiscus, with additional records identified through other sources e.g., Google Scholar (Table 1). Medical subject headings (MESHs) or title/abstract spelling terms and synonym variations were modified to suit each database. For the Scopus database, we performed separate title then abstract searches and used the capitalised ‘OR’ operand. Main headings used in Google Scholar, google searches and the University of Newcastle library.

### 2.2. Study Selection Process

**Figure 1:**
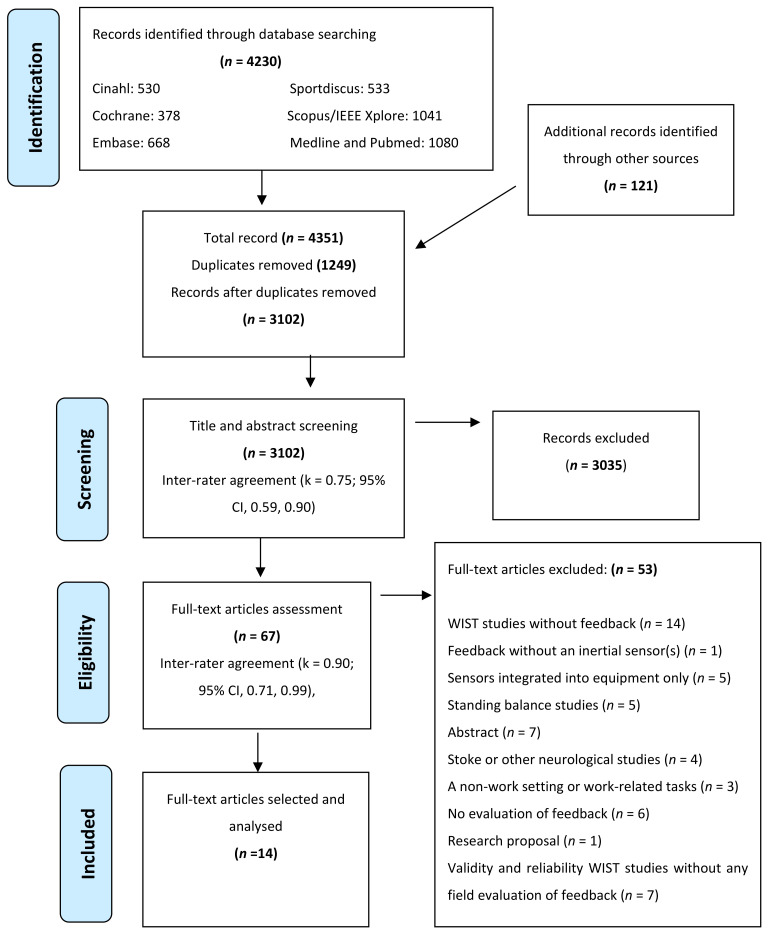
PRISMA diagram of the study selection process.

The selection process was reported in accordance with the PRISMA guidelines [59] (Figure 1).

Eligible studies were required to meet all four inclusion criteria: (1) the use of a WIST system to monitor or track upper body posture and/or movement behaviour using an on-body accelerometer or gyroscope or magnetometer, used in combination or individually, using real-time monitoring and the provision of feedback during work in a workplace setting or during work-related activities; (2) studies that report on feedback from WIST devices in individuals 18–65 years of age of any gender with or without an upper body musculoskeletal disorder (MSD); (3) peer-reviewed journal articles (or full engineering conference proceeding articles) that met criteria 1 and 2, irrespective of study quality; (4) articles in English with inclusion dates ranging from January 2005 (due to WIST being a relatively new technology) to July 2021. Data pertaining to device wearability and the use of WIST to quantify kinematics were extracted from studies that met these four inclusion criteria. Studies were not eligible that included movement theory, model-based movement or animal investigations. Studies of activities other than those at work or that were workplace-related (e.g., the activities of daily living or in-patient settings) were not eligible. Studies including neurological disorders (e.g., stroke or stroke rehabilitation) or conditions other than musculoskeletal disorders were excluded. Lower limb or standing balance studies using feedback from WIST were excluded to allow discussion of specific aspects related to neck and back MSD. Furthermore, studies that reported on validity, reliability or biomechanical evaluations were not eligible. Following the completion of computerised database searches, the removal of duplicates was completed by one investigator (RL) using the Endnote X8 citation manager [60] with any remaining duplicates detected and removed automatically using Covidence systematic review software [61]. Two investigators (RL and JY) independently screened articles. Disagreements following each screening round were resolved by consensus, or if consensus was not achieved consultation with a third investigator (SS). The level of inter-rater agreement between investigators for title/abstract and full text screening was assessed using Cohen’s kappa [62].

### 2.3. Data Extraction

One investigator (RL) independently organised and extracted data from the included studies, with accuracy checked by a second investigator (JY). The extracted data included the study characteristics: year, setting, study population and condition, study design, objective and comparison groups; the effect of WIST feedback; the technical characteristics of feedback: monitoring duration, type of feedback, feedback trigger, feedback source, origin of set-point and anatomical monitoring; and WIST system characteristics: model/manufacture, frequency, sensor type, sensor quantity, connection and attachment method, sampling rate, filter type, cut-off frequency, algorithm origin, sensor validation, anatomical location and device limitations.

### 2.4. Methodological Quality

The methodological quality of the included studies was assessed using the National Institutes of Health (NIH) risk-of-bias tools for ‘Observational Cohort and Cross-Sectional Studies’ (for cross-sectional studies), the tool for ‘Before-After (pre-post) Studies With No Control Group’ (for pre-post studies) and Controlled Intervention Studies (for randomised controlled trials) [63]. Two investigators (RL and JY) independently assessed the risk of bias; if a consensus was not met, resolution was achieved through consultation with a third investigator (SS). Cohen’s kappa was used to assess the level of agreement between reviewers. To control for rater bias, individuals were from different disciplines and detailed inclusion and exclusion criteria were used.

### 2.5. Quality of Evidence for the Effectiveness of Feedback

A synthesis to evaluate the levels of evidence was performed since the included studies were heterogenous in terms of the equipment detailed and study design. The synthesis rates the level of evidence for the reported outcomes from studies based on the following hierarchical criteria, as previously described [64,65,66]:

Strong evidence—consistent findings among three or more studies, including a minimum of two high-quality studies.

Moderate evidence—consistent findings among two or more studies, including at least one high-quality study.

Limited evidence—findings from at least one high-quality study or two low- or moderate-quality studies.

Very limited evidence—findings from one low- or moderate-quality study.

Inconsistent evidence—inconsistent findings among multiple studies (e.g., one or multiple studies reported a significant result, whereas one or multiple studies reported no significant result).

Conflicting evidence—we defined conflicting as contradictory results between studies (e.g., one or multiple studies reported a significant result in one direction, whereas one or multiple studies reported a significant result in the other direction).

No evidence—results were insignificant and derived from multiple studies regardless of the quality.

## 3. Results

A total of 4351 articles were identified from the databases and additional record searches (Figure 1). Duplications (1249) were removed. Title and abstract screening of 3102 studies excluded 3035 studies, with most exclusions based on no feedback and/or evaluation, reliability and validity studies, abstracts, or a lack of relevance to upper body posture or work activities. Following the full-text screening of 67 studies, a further 53 studies were excluded (reasons provided in Figure 1 and Section 3.1). Fourteen studies met the inclusion criteria of this review. The characteristics of the included studies are summarised in Table 2.

### 3.1. Excluded Studies

A total of 53 studies were excluded in full-text screening as follows: WIST studies without feedback (*n* = 14) [55,81,82,83,84,85,86,87,88,89,90,91,92,93]; feedback without an inertial sensor (s) [94]; sensors integrated into equipment (*n* = 5), e.g., seat sensors and robotic devices rather than those worn by an individual [95,96,97,98,99]; standing balance and/or lower body sway (*n* = 5) [100,101,102,103,104]; abstracts (*n* = 7) [105,106,107,108,109,110,111]; stroke/other neurological rehabilitation studies (*n* = 4) [112,113,114,115]; a non-work setting (*n* = 3) [116,117,118]; no evaluation of WIST feedback effectiveness (*n* = 6) [119,120,121,122,123,124]; research proposal (*n* = 1) [125]; and validity and reliability studies without a field assessment of WIST feedback (*n* = 7) [40,42,126,127,128,129,130].

### 3.2. Effectiveness of Feedback

The majority of studies reported improvements in primary outcomes using feedback compared to no feedback with no negative health effects (Table 3). No included study reported post-intervention monitoring to assess the retention of improvements following WIST feedback. Four types of feedback prompts were identified throughout the included studies: auditory [68,71,73,74,75,80]; vibrotactile (haptic) [69,72,74,76,77,78,79]; visual [67,70,71,72,74,75,78] and summary feedback (visual) [74]. The most common multimodal feedback interaction was auditory and visual [71,74,75]. Most studies applied concurrent bandwidth feedback [67,69,73,75,76,77,79,80] (i.e., a feedback prompt when a movement variable exceeds a pre-determined set-point (feedback trigger) during the activity/task [131,132]; and in conjunction with a pre-determined time period [68,71,72,74,78] (Table 4); the remaining studies used terminal bandwidth feedback (feedback post-activity) [70] and summary feedback in addition to visual, auditory and vibrotactile feedback [74].

Improved trunk posture occurred using various types of WIST feedback for different tasks, for example, auditory during lifting [73], moving patients from bed to chair [80] and office tasks [68], vibratory/auditory during nursing tasks [74], visual/vibrotactile during several simulated workplace tasks [78] vibrotactile during a computer task [76] and vibrotactile [69] and visual [70] during dental procedures (Table 3). However, using vibratory feedback alone during sedentary tasks resulted in no trunk posture improvements [77]. Improved neck posture was observed using WIST feedback: vibrotactile/visual [72,78] and visual/auditory [75] and vibratory during computer tasks [76], and visual during a dental procedure [70]. Visual/auditory feedback reduced the exposure to WMSD during an industrial task [71]. A slight risk increase (RULA/LUBA) was observed for arms during tasks 2–4 using visual/vibrotactile feedback [78] (though the results were confounded by participants reaching for the chair during these tasks). However, [79] identified less accumulated time and angles for arms during the simulated mail sorting tasks. Visual feedback increased step counts during office tasks [67]. Two studies reported changes in pain symptoms from WIST feedback: increased neck pain during a computer task [76], and no significant reduction in lower back pain during sedentary work [77] (Table 3).

**Table 3:** Effect of wearable inertial sensor technology (WIST) feedback on participant outcomes in each of the included studies.

Study	Reported Effect from Feedback	
Brakenridge, Fjeldsoe [67]	Improved between-group differences in movement behaviour at 12 months in overall hours/16 h using feedback compared to no feedback: Increased stepping time: +20.6 min (95% CI, 3.1, 38.1), *p* = 0.021 Increased step count +846.5 steps (67.8, 1625.2), *p* = 0.003 Improved within-group differences from baseline to 12 months during work hours/10 h using feedback compared to no feedback: Increased stepping: +9.1 min (0.2, 17.9), *p* = 0.045	
Ribeiro, Sole [68]	Reduced rate within-groups of exceeding lumbar (lower back) postural threshold using constant feedback compared to intermittent and no feedback (4-week follow-up minus baseline): Constant feedback: frequency/h −0.9 (95% CI, −1.9, −0.1), d = 51 *p* = 0.03 Large effect between-group postural patterns favoured constant feedback (4-week follow-up minus baseline): Constant feedback: frequency/h −0.49 (−1.62, 0.64), d = 0.60, *p* = 0.91	
Thanathornwong and Suebnukarn [69]	Decreased upper trunk flexion and lateral trunk flexion using feedback compared to no feedback: flexion 3.6° to 8.5° (95% CI, NR) *p* = 0.05 lateral flexion 6.1° to 8.9° (95% CI, NR) *p* = 0.05	
Thanathornwong, Suebnukarn [70]	Decreased flexion using feedback compared to no feedback: Mean (SD) Neck: pre-test: 16.7° (8.88); post-test 10.5° (7.29) *p* < 0.05 Upper trunk: pre-test: 22.0° (6.1); post-test 12.8° (6.58), *p* < 0.05	
Vignais, Miezal [71]	Reduced risk of WMSD between-group for lower global RULA † scores using feedback: Mean (SD): Feedback 3.95 (.83); no feedback 4.35 (.54), *p* < 0.05 Decreased time spend in each RULA range using feedback: % (SD) Range 3–4 feedback 76.4% (17.7); no feedback 56.9% (13.6), *p* < 0.05 Range 5–6 feedback 16.8% (13.2); no feedback 30.5% (6.9), *p* < 0.05 Range 7 feedback 3.4% (5.5); no feedback 10.4% (12.2), *p* = 0.07 Decreased neck exposure to hazardous posture: % (SD) Feedback 12.24% (15.89); no feedback 34.03% (10.8), *p* < 0.05 Overall time in task: Mean (SD) seconds Feedback 227.9 s (33.7); no feedback 157.0 s (28.9), *p* < 0.005	
Ailneni, Syamala [72]	Reduced cranio-cervical and neck flexion angle during sitting computer condition favouring feedback: Mean, (SD) Neck angle: Feedback 57.52° (1.25); no feedback 63.16° (1.83), *p* = 0.02 Cranio-cervical angle: Feedback 157.14 (1.89); no feedback 160.90 (2.00), *p* = 0.01 No significant difference between head flexion Reduced cranio-cervical and neck flexion angle during standing computer condition favouring feedback: mean, (SD) Neck angle: feedback 58.49° (1.11); no feedback 63.21° (1.38), *p* < 0.01 Head angle: feedback 81.32 (2.01); no feedback 84.35 (1.69), *p* = 0.04 No significant difference between Cranio-cervical angle	
Boocock, Naudé [73]	Decreased lumbar (lumbosacral) flexion at 20th minute: Feedback 182.6° (95% CI, 182.6–190.4); no feedback 188.2° (182.7, 193.8), *p* < 0.001 Decreased trunk flexion at 20th minute: Feedback 27.4° (23.7–31.1); no feedback 48.3° (43.5, 53.2), *p* < 0.001 Time to perform lift (s) at 20th minute: feedback 1.07 s (0.99, 1.14); no feedback 1.31 s (1.17, 1.45), *p* = 0.01	
Bootsman, Markopoulos [74]	Improved lumbar posture occurrences reduced using feedback compared to no feedback: mean, (SD) Feedback (vibration and audible) M = 22.1, (10.8) and feedback (vibration, audible and smartphone) M = 19.1, (12.2); no feedback (baseline) M = 25.5 (12.5); no feedback (withdrawal condition) M = 24.9, (12.8). No significant between feedback conditions	
Breen, Nisar [75]	Reduced time spent in poor neck (flexion/extension) posture using feedback during a 5-h period: Feedback 6.5% (SD, 9.6); no feedback 35.7% (15.26), *p* < 0.05	
Kuo, Wang [76]	Between-group difference favouring feedback compared to no feedback Reduced neck flexion 3.3° (95% CI, 1.8°, 4.7°), *p* < 0.001 Reduced upper cervical angle 3.3° (1.7°, 5.0°), *p* < 0.001 Reduced lower thoracic (lumbar) angle 1.6° (0.4°, 2.7°), *p* = 0.001 Increased NRS score between-group difference: ↑ time ↑ neck pain: 1.6 (0.9, 2.4), *p* < 0.001 ↑ time ↑ shoulder pain: 1.8 (1.0, 2.7), *p* < 0.001 Decreased cervical erector spinae activity: Right 24.9% (8.4, 41.5), *p* = 0.005; left 24.6% (7.7, 41.5), *p* = 0.007	
Park, Hetzler [77]	No between-group difference in Cornell musculoskeletal discomfort questionnaire scores (CMDQ): Lower back pain (LBP) experience: (F (1,29) = 0.58, *p* = 0.45 LBP discomfort (F (1,18) = 0.14, *p* = 0.71 LBP interference (F (1,18) = 0.93, *p* = 0.35 No relationship between number of good posture hours and CMDQ score changes: LBP experience r^2^ (0.17), *p* = 0.28 LBP discomfort r^2^ (0.03), *p* = 0.87 LBP interference r^2^ (0.28), *p* = 0.20)	
Cerqueira, Da Silva [78]	Reduced HR (high risk) level for neck using feedback compared to no feedback: task 2: 36.6%, task 3: 43.6%, task 4: 45%, and task 5: 26% Reduced HR (high risk) level for trunk using feedback compared to no feedback: tasks 1–5 respectively 1.8%, 22.4%, 39.8%, 28.6% and 4.6% No HR (high risk) level for arms using feedback compared to no feedback during all 5 tasksLonger task duration using feedback (M = 343.98 ± 47.27 s) without feedback (M = 263.98 ± 46.47 s)	
Lind, Diaz-Olivares [79]	Less accumulated time (difference %) and angle (difference %) in upper-arm elevations using feedback compared to baseline (no feedback)	
	Feedback 1 (accumulative time): ≥30° (↓38%) *p* = <0.001 ≥45° (↓36%) *p* = <0.001 ≥60° (↓49%) *p* = 0.001 Feedback 2 (accumulative time): ≥30° (↓29.7%) *p* = <0.001 ≥45° (↓14%) *p* = <0.001 ≥60° (↓4.5%) *p* = <0.001	Feedback 1 (elevation angles): 50th (↓32%) *p* = <0.001 (↓16%) *p* = <0.001 (↓10%) *p* = 0.002 (↓13%) *p* = 0.001 Feedback 2 (elevation angles): 50th (↓33%) *p* = <0.001 (↓21%) *p* = 0.001 (↓19%) *p* = 0.001 (↓16%) *p* = <0.001
Doss, Robathan [80]	The bed-to-chair condition using feedback compared to no feedback reached significance *: Decrease in mean time to complete each task 6.2° (4.4) second or 23.3% decrease *p* = 0.01, reduction in trunk flexion 7.6° *p* = 0.05 Reduction in peak trunk flexion/extension (flexion = 1548 (38)°/S2 (*p* = 0.01) representing a 46.9% decrease) (extension = 1020 (74)°/S2 (*p* = 0.03)) Peak lateral bending acceleration right reduced 1189 (39)°/S2 38.3% decrease (*p* = 0.01) and left reduced 1473 (187)°/S2 48.4% decrease (*p* = 0.0007) Reductions in peak rotation acceleration left 1188 (143)°/S2 (*p* = 0.003), right 1398 (1.3)°/S2 (*p* = 0.001) Reduction in time to complete task 6.2 (4.4) seconds (*p* = 0.01) * Significance not reached for conditions using a sling under and/or patient adjustment	

NR: not reported; † RULA: The Rapid Upper Limb Assessment [133].

**Table 4:** Technical characteristics of wearable inertial sensor technology (WIST) feedback in each of the included studies.

Study	Monitoring Duration (h/min)	Type of Feedback	Feedback Trigger (Set-Point)	Feedback Source	Origin of Kinematic Set-Point	Anatomical Monitoring/Direction
Brakenridge, Fjeldsoe [67]	Self-directed use. >1 h = valid day. 12-month intervention	Visual (concurrent)	Device app compares initial daily calibration ¥	Smart phone	Manufacturer	Sagittal plane: Lumbopelvic (flexion/extension)
Ribeiro, Sole [68]	4 weeks: working hours only. Mean h (SD): 5.9 (1.9)	Auditory (concurrent with latency)	Exceeding cumulative ROM threshold: Feedback triggered when workers exceed 45° pelvic flexion + max of 2° flexion/min +static posture (flexed pelvis) = 5 s	Sensor device	Literature-based	Sagittal plane: Lumbopelvic: (flexion/extension)
Thanathornwong and Suebnukarn [69]	NR.	Vibrotactile (concurrent)	Exceeding posture outside the norm of the hidden Markov models (HMMs)	Sensor device	Hidden Markov models (HMMs)	Sagittal and frontal plane: upper body (lateral flexion; flexion/extension)
Thanathornwong, Suebnukarn [70]	NR.	Visual (terminal)	Exceeding posture outside the norm of the hidden Markov models (HMMs)	NR	Hidden Markov models (HMMs)	Sagittal and frontal plane: upper body and head (lateral flexion; flexion/extension)
Vignais, Miezal [71]	4 min	Visual (incorporated in to STHMD) and auditory (concurrent with latency)	Auditory: RULA global score = 7, => 0.5 s; 5–6, =5 s Visual: Local score: Shoulder and upper arm > 5; Elbow and lower arm >3; Wrist and hand >5; Neck and head > 4; Pelvis and trunk > 4. ¤	Within the head-mounted display	Rapid Upper Limb Assessment (RULA)	Sagittal, frontal and transverse plane: upper body (lateral flexion; flexion/extension and rotation)
Ailneni, Syamala [72]	2 h	Vibrotactile and visual (concurrent with latency)	Neck flexion angle greater than 15° and exceeding 30 s relative to neutral posture ¤	Sensor device	Literature-based	Sagittal plane: neck/head posture (Flexion/extension)
Boocock, Naudé [73]	20 min	Auditory (concurrent; high pitched tone)	80% of maximum lumbosacral range of motion was exceeded ¤	Purpose-built software	Literature-based	Sagittal plane: lumbosacral, trunk posture (Flexion/extension)
Bootsman, Markopoulos [74]	4-phase treatment: baseline 30 min; per phase A, B and C = 60 min each. Total duration 210 min	Auditory, vibrotactile, visual and summary feedback (concurrent with latency)	>20° from neutral posture during lower back flexion and exceeding 1.5 s	Garment (auditory and vibrotactile) Visual (smartphone)	Literature-based	Sagittal plane: lumbar spine (Flexion/extension)
Breen, Nisar [75]	5 h without feedback, another 5 h with feedback	Visual and auditory (concurrent)	Exceeding −5 to 10° threshold	Visual to user via a graphical interface (GUI) on a computer	Literature based	Sagittal plane: neck cranial-vertebral: (flexion/extension).
Kuo, Wang [76]	2 h	Vibrotactile (concurrent)	Exceeding threshold ¥	Sensor device	Manufacturer	Sagittal plane: trunk posture (Flexion/extension)
Park, Hetzler [77]	21 days during working day (8.5 h average per day)	Vibrotactile (concurrent)	Exceeding threshold ¥	Sensor device	Manufacturer	Sagittal and frontal plane: Upper body posture
Cerqueira, Da Silva [78]	Maximum duration 391 s (<6.5 min)	Visual and vibrotactile (concurrent)	Combination of RULA and LUBA thresholds Trunk sagittal: (risk) (high) < −10° ∆t > 1 s extension (high) > 60° ∆t > 1 s flexion (medium) <20° <60° ∆t > 10 s flexion (low) −10° <20° desirable Trunk coronal: (risk) (medium-high) < −10° or >10° ∆t > 5 s bent left or right (low) −10° <10° desirable Neck sagittal: (high) <−5° ∆t > 1 s extension (high) >20° ∆t > 1 s flexion (medium) 10° <20° ∆t > 10 s flexion (low) −5° <10° desirable Neck Coronal: (medium-high) <−5° or >5° ∆t > 5 s bent to left or right (low) −5° <5° desirable Arm sagittal: (high) >90° ∆t > 1 s (medium-high) <−20° ∆t > 5 s shoulder adducted (medium-high) 45° <90° ∆t > 5 s abducted (medium) 20° <45° ∆t > 10 s (low) −20° <20° desirable Arm coronal: (medium-high) −20° or >20° ∆t > 5 s shoulder flexed/extended (low) −20° <20° desirable	Haptic motors × 4 and visual to user via a graphical interface (GUI) on a computer	Literature based on rapid upper-limb assessment (RULA) and loading on the upper body (LUBA)	Sagittal and coronal plane of the trunk, neck and arm.
Lind, Diaz-Olivares [79]	<15 min	Vibrotactile (concurrent)	Exceeding ≥30° and ≥60° threshold for the dominate arm	On-body two-frequency-level vibrotactile unit	Literature-based	Sagittal plane: upper arm flexion
Doss, Robathan [80]	NR	Auditory (concurrent)	>45° trunk flexion	Smart phone	Literature-based	Sagittal plane: trunk posture (flexion)

NR: not reported. ¤ Researcher discretion; ¥ manufacturer discretion: all biomechanical set points/thresholds are pre-determined by the researchers or manufacturer. STHMD: see-through head-mounted display. Min: minutes.

### 3.3. WIST Device Wearability

The sensor attachment methods in the fourteen included studies were diverse: ‘on’ clothing (*n* = 6) [68,69,70,78,79,80]; within a smart sensing garment (*n* = 1) [74]; worn as a belt (*n* = 1) [67]; direct skin attachment using tape (*n* = 2) [73,76]; magnetic clasp to undershirt (*n* = 1) [77]; secured by bands on ears positioned posteriorly on neck [72]; and not reported (*n* = 2) [71,75] (Table 5). Ribeiro, Sole [68] evaluated workers’ perception of WIST usefulness using a Likert scale. Three studies (*n* = 3) [74,78,79] provided a comprehensive evaluation of their WIST device (garment) in terms of user comfort and acceptability, e.g., three validated questionnaires and a semi-structured interview [74], assessments of users experience via a semi-structed interview and discomfort/pain using the Borg CR10 [134] scale [79], and Cerqueira, Da Silva [78] applied the guidelines of the System Usability Scale (SUS) [135].

### 3.4. Use of WIST Systems to Quantify Kinematics

A tri-axial accelerometer was used in all included studies, with the tri-axial IMU used in five studies [71,73,74,78,79] (Table 5). On-body sensor quantities were diverse between studies, e.g., IMU studies (*n* = 5) [71,73,74,78,79] ranging from two IMUs to seven on-body sensors. Increasing the number of IMUs enabled greater complexity in movement data within a three-dimensional (3D) space. The remaining studies [67,68,69,70,72,75,76,77] (*n* = 8) applied one sensor. Three studies reported a rationale for sensor quantities [74,75,78]. Eight studies developed custom WIST systems and/or software [69,70,71,74,75,78,79,80] to address their specific research; the remaining studies [67,68,72,73,76,77] utilised commercial devices.

Sensor sampling frequency and data processing methods (filtering type and filtering cut-off frequency) were not reported in studies using a commercial device nor in some customised studies [67,68,69,70,72,73,74,76,77,80]. Three studies [71,75,78] reported the sensor sampling frequency; two studies reported the sampling frequency range [69,70]. The reported limitations of WIST were sensor drift [67,79]; a lack of time stamping during data recording [68]; inconsistencies in Bluetooth connection [77,79]; software issues [69]; a lack of degrees of freedom (DOF) [75]; loose fitting sensors [70]; magnetic material interference with the magnetometer signal [71]; garment may not suit individual anthropometric measurements [74]; and a potential reduction in sensitivity without direct validation [72,79] (Table 5).

Two of the included studies conducted a prior evaluation into WIST system reliability and validity [79,136]; another study validated the WIST system prior to use [78] (Table 5). No other studies reported on the reliability nor validity of their WIST system [69,70,71,72,74,75,80]; the remaining included studies reported or mentioned the validation results from the manufacturer [67,73,76,77]. A three-dimensional motion capture system was used simultaneously with the WIST sensor(s) in four studies [72,73,76,80].

### 3.5. Risk of Bias

Inter-rater agreement between investigators (RL and JY) was high: title and abstract screening (k = 0.75; 95% CI, 0.59, 0.90); full text screening (k = 0.90; 95% CI, 0.71, 0.99). Controlled intervention studies (*n* = 2; Table 6) scored well in terms of the study description, the sample size being sufficient to detect differences and randomisation. Bias was identified in several quality criteria, e.g., blinding, baseline characteristics, dropout rates, adherence protocols and outcomes being valid and reliable. Before–after studies (*n* = 2; Table 7) scored well in terms of their stated objectives, sample size, intervention description and statistical tests used in outcome measures, with bias identified in their participant eligibility criteria, validity and reliability in reported outcomes. Observational cohort and cross-sectional studies (*n* = 10; Table 8) scored poorly to fairly, as certain criteria were absent from several studies. The risk of bias assessment categorised five studies (*n* = 8) as ‘fair’ and the remaining six studies (*n* = 6) were categorised as ‘poor’ (Table 6, Table 7 and Table 8).

### 3.6. Quality of Evidence

**Figure 2:**
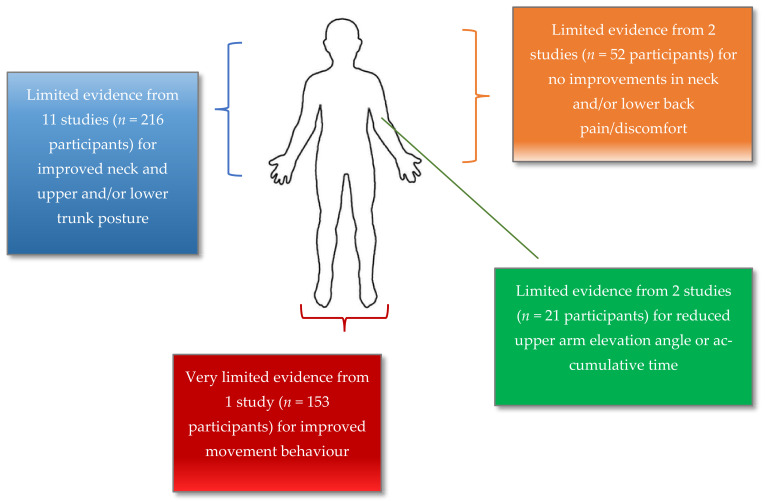
Overview of evaluated evidence for the effects of feedback from wearable inertial sensor technology on upper body posture and movement behaviour during workplace-related tasks.

The synthesis of the quality of evidence supporting WIST feedback (Table 9, Figure 2) identified a ‘limited’ level of evidence from eleven studies to support improvements in neck and upper and lower trunk posture; ‘limited’ evidence from two studies supporting improved neck and lower back pain/discomfort; ‘very limited’ evidence from one study supporting movement behaviour; and ‘limited’ evidence from two studies to support a reduction in upper-arm elevation angle or accumulative time. Many included studies were not forthcoming in details about WIST technology/equipment, study design, sensor validation or data collection procedures; hence, methodological reproducibility would not be achievable. Therefore, to improve the consistency and quality of the evidence of future WIST studies, in this review we propose a ‘Technology and Design Checklist’ (TDC) to improve on the minimum reporting criteria (Table 10). The TDC is a checklist for researchers of the essential technical and study design aspects to consider reporting when designing a study using WIST. The objective of the TDC is to support future research investigating the effects of WIST, to minimise reporting omissions.

## 4. Discussion

This review provides evidence for the effectiveness of feedback from WIST for work or work-related activities. The review summarises the effects of WIST feedback on upper body kinematics and movement behaviour, then discusses wearability and the use of WIST to quantify kinematics, as expressed in the fourteen included studies. Meaningful and clinically relevant improvements in posture and/or movement behaviour were observed using WIST feedback compared to not using feedback (Table 3), although no improvements in pain symptoms were identified. The duration of feedback was diverse, ranging from 4 min [71] to 12 months [67]. Longer interactions of feedback may improve the retention of learnt skills [48], but no included study investigated the effects of varying durations. Visual and/or vibrotactile (haptic) feedback were the most applied feedback strategies (Table 5). Only three included studies assessed wearability to indicate the level of device acceptability, and most of the included studies did not comprehensively report on WIST technical aspects or device validity (Table 5). Of the fourteen included studies, tri-axial accelerometers, followed by IMUs, were the most frequently used technologies (Table 5). This review identified lower levels of evidence in supporting any of the identified outcomes resulting from WIST usage, due to poor/fair study quality and between-study heterogeneity, preventing data pooling.

### 4.1. Effectiveness of Feedback Strategies

Overall, most of the reported outcomes from the use of WIST feedback assessed in the current review were positive, i.e., improved upper body posture or movement behaviour in users. WIST feedback can have practical merit in the workplace, where real-time feedback is a constant reminder of adverse posture and/or movement behaviour compared to previously learnt ergonomic instruction that tends to be forgotten, especially during cognitively demanding activities [138]. However, gauging the effectiveness of a particular feedback type was difficult due to the between-study heterogeneity of tasks evaluated and feedback strategies used (Table 3). The effectiveness of various feedback types has been previously debated in motor relearning interventions that reported varying success [139,140,141]. Visual and/or vibrotactile feedback were the most commonly preferred feedback strategies in this review. Visual feedback was rarely used individually (*n* = 2) and may be paired with auditory feedback (*n* = 2) or vibrotactile (*n* = 2) feedback. Only one included study applied three feedback strategies [74]. Combining visual and auditory feedback is more effective than using visual feedback alone to improve performance during a single task [142], which might explain their use (*n* = 3) among studies within this review.

Vibrotactile and/or auditory feedback strategies do not require visual attention, which may be preferred for some tasks that require constant visual attention. However, visual feedback can enhance users’ learning through visualising their movement with greater detail, and is commonly applied in upper body rehabilitation [43]. Audible feedback in a workplace environments may not be practical, and can incur potential confounding effects; for example, users may become self-conscious or embarrassed during audible feedback, which may adversely affect their task performance [74], or feedback may become dampened due to a noisy environment [78]. Hence, any type of WIST feedback should be suitable for that working environment and should not distract the user or others from their tasks.

Concurrent bandwidth feedback was the preferred method of feedback interaction in most included studies (*n* = 13/14 studies) (Table 4), and this is consistent with other postural and rehabilitation reviews [43,103,131,139]. The consensus suggests that feedback content should match the user’s proficiency to the specified task, for example, concurrent bandwidth feedback is most suited to non-proficient users for shorter feedback periods, whereas individuals with higher skill levels are suited to terminal feedback [43,131], as applied during a dental procedure in one included study [70], and/or for longer training periods [79]. A pre-determined latency period is often incorporated to prevent the excessive prompting of feedback during short-term aberrant movements [143]. Latency was applied in the feedback strategies of several of the included studies to assist with any unnecessary prompting (*n* = 6) [68,71,72,74,78,79]. These examples suggest that the selection of feedback types/schedules are dependent on the task and environmental constraints [144]. This review identified no study that applied feedback fading or self-controlled frequency schedules to reduce an individual’s dependence on feedback within a given task [131,141].

### 4.2. Effects of WIST Feedback on Posture, Movement Behaviour and/or Pain

Our review findings are consistent with other recent reviews that have identified that feedback from WIST can be effective. However, the majority of reviews focused on the use of WIST feedback in sporting applications, balance or stroke [44,145,146]. Wang, Markopoulos [43] reviewed 45 studies using WIST for rehabilitation and found only three studies that reported the clinical effects of WIST feedback, primarily in populations with stroke. One review that examined the effects of feedback from devices other than inertial sensors found moderate evidence that feedback from surface electromyography (sEMG) does not prevent WMSD [23]. Another found that feedback from a computer mouse caused workers to modify their postures, which resulted in the reduction of neck and/or shoulder WMSD in workers [147]. In the majority of reviews, the authors appeal for higher-quality studies investigating WIST feedback [44,146,148]. Thus, improving the quality of future studies may enable the greater utility of WIST in rehabilitation, clinical and workplace settings.

In the current review, all the included studies that reported feedback from WIST compared to no feedback demonstrated improved upper body postures (reductions in non-neutral positions) and/or movement behaviour (Table 3). In this review, ‘very limited’ evidence from one study was identified to support changes in movement behaviour from WIST feedback. Improvements in upper body posture and/or movement behaviour can be learnt rapidly using feedback from WIST [73,76]. However, retaining learnt behaviour post-feedback intervention is suggested to be more dependent on the duration of the feedback interaction than the content/type of feedback (visual, vibrotactile, audible or multimodal) [48]. For example, compared to baseline or 3-month follow up, the included study by Brakenridge, Fjeldsoe [67] identified significant improvements in movement behaviour using WIST feedback at the 12-month period. In contrast, the included study by Bootsman, Markopoulos [74] found that participants immediately reverted to baseline postures during no WIST feedback despite improved posture during the previous 60-min multimodal WIST feedback phase (Table 4), or if participants retained knowledge of improved lifting tasks post-feedback intervention [80]. This may suggest that feedback distributed across a greater time period is more effective at modifying behaviour and causing acceptance in learning than feedback delivered during a single point in time [149]. Nevertheless, only one study in this current review reported on the longevity of feedback [67]. In other sectors of health research, the retention of learned movement behaviour from WIST was shown for arm-hand movement in stroke rehabilitation [43,150] and lower limb running biomechanics [44]. Previous research on the retention of skills following feedback has indicated that a fading schedule of feedback is most effective for motor-relearning and for learned skills to be retained, suggesting that gradually reducing the dependence on external feedback improved the intrinsic feedback mechanisms and subsequent motor re-learning to occur [44,132,151]. Though, the retention of skills following feedback is seldom evaluated [146]; hence, further post-evaluation research is required.

This review identified ‘limited’ evidence from two work-related studies that WIST feedback does not improve neck and lower back pain/discomfort (Table 9). Despite improved posture as a result of WIST feedback, participants in two studies reported pain (increased neck pain [76] during a one-hour task, and no significant change in lower back pain [77] during a three week intervention) (Table 3). However, pain reduction may not be immediately evident using WIST feedback; for instance, previous research found that lower back pain symptoms subsided near the end of the six-week intervention [152]. Kent, Laird [48] identified that the WIST feedback group self-reported a slight peak in lower back pain at the 8-week mark, followed by a clinically relevant difference in pain reduction at the 3-month and 12-month follow-up compared to a control. This suggests that pain may worsen initially until an individual adapts to their new postural state. This circumstance may occur as individuals with neck or lower back pain are more likely to experience maladaptive neuromuscular control, which may require longer periods in rehabilitation [153,154,155], which may challenge postural changes in response to short-term feedback strategies. Analogous to improvements in posture or movement behaviour, the likelihood of retaining learnt behaviour to reduce pain appears to be dependent on longer periods of feedback interaction. However, extrinsic feedback dependency may arise from a longer duration of concurrent feedback dominance, causing the user to be less responsive their body’s own internal or intrinsic feedback mechanisms [132]. Hence, WIST feedback latency during rehabilitation studies must be considered.

This review identified ‘limited’ evidence from eleven studies that WIST feedback improves neck, upper and lower back posture (Table 9). The included studies investigating lower back kinematics reported results indicating that feedback from WIST improved lumbar posture during sitting (1.6°) [76], reduced trunk flexion during patient bed-to-chair transfers (7.6°) [80] and resulted in clinically relevant changes in lumbar tilt during a lifting task (15.2°) [73]. These findings are consistent with a previous study on the activities of daily living showing a reduction in lumbar flexion (~23°) from WIST feedback [36]. However, variations in joint angle magnitude can be due to differences in inter-segmental angle definition, participant demographics or activity requirements. Feedback triggers (kinematic set points) were heterogenous between studies (Table 4). Therefore, the determination of an average value for changed postures from the included studies was unachievable, e.g., triggers for postural change occurred when exceeding 45° lower back flexion for longer than five seconds [68], greater-than-45° trunk flexion without latency [80];,greater-than-20° lumbar flexion for 1.5 s [74] and exceeding 80% of the maximum lumbar range of motion [73]. Nevertheless, as neck and lower back pain are a leading cause of global disability [5,156], changes in posture from WIST feedback that may reduce the risk of WMSD are encouraging. Greater magnitude in neck flexion is associated with an increased risk of the development of neck pain [157], especially during prolonged computer use [158]. Additionally, individuals that adopt a forward head posture (large cranio-cervical angle in the sagittal plane) are more likely to experience neck pain [159] and pain-induced headaches [160]. Three included studies showed a significant reduction in neck flexion that ranged between 3° and 6° using WIST feedback compared to no feedback during computer use [72,76], and during a dental procedure [70]. In previous research, individuals with neck pain presented with 6.8°-greater neck flexion compared to asymptomatic individuals [161]. A reduction in the gravitational moment of the neck joint [162] may assist in pain reduction [157], muscular fatigue and lower MSD risk [163]. Another three studies in this review [71,75,78] showed significantly less time spent in ‘adverse neck postures’ using feedback from WIST, suggesting that ‘less hazardous’ postures were adopted during the task using WIST feedback compared to no feedback. Though industrial processes have automated some repetitious workplace activities, manual handling tasks are in most instances still a feasible and viable option for many businesses to adopt [164]. Hazardous postures may be dependent on the actual task undertaken, for example, the included study by Lind, Diaz-Olivares [79] identified a significant reduction in adverse (high-risk) upper arm positioning, although the participants in the study by Cerqueira, Da Silva [78] did not present any arm postures in the high-risk category, as determined by RULA or LUBA guidelines. Despite these included studies having promising outcomes, the level of evidence for improved upper body posture was limited; therefore, caution during interpretation is recommended.

### 4.3. Device Wearability

Wearability guidelines consider appropriate sensor placement to enhance user comfort, device usability [45] and device accuracy [165]. Therefore, a single sensor or wireless body area network (WBAN) design must consider conforming to the user’s body, weight, attachment method, connection (data transmission, wireless/cable), interaction with movement, unobtrusiveness, duration of use and thermal aspects (breathability between skin and the device) [46]. The placement/attachment of individual sensor(s) were only superficially described throughout most of the included studies. The included study by Bootsman, Markopoulos [74] used an e-textile garment integrated two tri-axial IMUs into the workplace uniform, providing greater comfort and practicality compared to other common methods of sensor placement (e.g., directly on skin) (Table 5). However, in the study by Bootsman, Markopoulos [74], accuracy was considered, though not assessed, suggesting that the garment may have introduced error if loosely fitted to the skin. Nonetheless, studies using e-textile garments have shown promising results in neurological rehabilitation [43,120,166], and thus further investigation of this approach during work-related activities is warranted.

To determine technology acceptance, wearability must also incorporate the user’s experience and perception of WIST feedback. The included study by Bootsman, Markopoulos [74] identified that wearability influenced device usability, with feelings of negative social influences being expressed by users when patients and/or colleagues overheard the audible feedback that emanated from the WIST garment during its use; which may potentially affect task performance [167]. Other areas of health and rehabilitation services have experienced similar issues when using audible feedback opposed to more subtle feedback strategies such as vibratory feedback [168,169]. Therefore, each sensor should not be salient or distract the user. Assessments in wearability (comfort, usability and safety [167,170]) are a benchmark for device improvements in future, especially for studies conducting prolonged monitoring [46]. However, in the current review, studies rarely addressed wearability, limiting the translation of WIST initiatives into practice.

### 4.4. Use of WIST Systems to Quantify Kinematics

The included studies indicated that the tri-axial accelerometer was used to track more simplistic body movements, whereas tri-axial IMUs tracked more complex kinematic movements, increasing the measured DOF during tasks (Table 4). Hence, sensor selection appears to be dependent on the complexity of the desired detection of movement during a specific task, which is consistent with other recent studies [30,36]. A known limitation is gyroscope drift [171], which occurs from accumulative measurement errors generated by fluctuating offset averages and measurement noise (despite appropriate calibration) [172] as reported in two included studies [68,79]. Additionally, magnetic disturbance can increase the divergence in yaw rotation accuracy (*z*-axis) in respect to time within the magnetometer signal [173,174]. These errors in orientation estimates can be mitigated through various filtration algorithms, e.g., the Kalman filter [175,176,177], and/or dedicated reference points, e.g., optical-based tracking systems integration [178]. Most included studies (*n* = 13) focused on less complex and dynamic movement rotations in flexion/extension (*x*-axis) and lateral flexion (*y*-axis) rather than head or body rotation (*z*-axis); therefore, orientation estimates were not affected by drift. To track complex movements, the included study by Vignais, Miezal [71] used multiple IMUs (9 DOF) to monitor rotations (*z*-axis) of the head, arm and upper trunk, and improved the level of certainty within the orientation estimates and the overall sensor accuracy by way of a Kalman filter [34,177]. However, the Kalman filter is not a fundamental requirement for all applications [174]. Nevertheless, differences in joint angles >10 degrees in magnitude with and without this filter during kinematic testing have been reported [172,179]. Understanding these limitations will help to improve reporting accuracies in future studies that track complex movements.

Many included studies did not disclose sampling frequency nor filtering cut-off frequency (Table 5). These are essential components to ensure WIST device accuracy, reliability and validity [180,181]. The Nyqusit sampling theorem may be violated if the sampling frequency is too low, as kinematic data may be lost in the sampling process [33,182]. Too low or high filtering cut-off frequencies will over-smooth the data or incur unwanted noise in the output data, respectively [33]. Although no reporting standards currently exist, failing to report these parameters reduces the overall level of confidence in the stated outcomes.

Importantly, WIST device validation against a gold standard such as a 3D motion capture system is paramount and is a requirement for successful translation into clinical practice [30,36,183]. Only the included study by Cerqueira, Da Silva [78] conducted a direct validation analysis to determine sensor accuracy, although Ribeiro, Sole [68] referred to their previous study, which assessed WIST device validity and reliability using a similar study design and setting (Table 5). As WIST device accuracy and reliability are task- and environment-dependent [30], achieving appropriate sensor validation for a specific task and location is a necessity. Thus, reported outcomes from the included studies without validation should be viewed with caution.

## 5. Study Limitations

This review is limited to the included studies that applied feedback from WIST for work-related tasks; therefore, examinations of device wearability and the use of WIST to quantify kinematics was summarised only from these 14 studies. We acknowledge that further studies on device wearability and the use of WIST to quantify kinematics exist; however, they were not the focus of this review. The included studies were heterogeneous in terms of workplace settings and activities, anatomical regions of interest, the level of WIST development and the reported outcomes. Hence, the pooling of data was not achievable. However, some studies have reported meaningful and clinically relevant differences using their specific WIST. No summary for wrist/hands nor for task duration comparing feedback to no feedback was conducted, as information in the included studies was scarce.

## 6. Future Research

A risk of bias and a lack of detail in reporting for methodological reproducibility was identified for most of the included studies. Therefore, in this review we propose a ‘Technology and Design Checklist’ for minimum reporting in studies evaluating outcomes using WIST or WIST interventions (Table 10). The checklist has four key research recommendations (data collection, WIST processing/analysis, feedback parameters and study design) to assist researchers in improving methodological quality in future studies. The reliability and validity of WIST should be reported to ensure dependability in reported outcomes. Future studies should investigate skill retention following WIST feedback. Additionally, greater collaboration between researchers and health professionals may assist in translating WIST more effectively into clinical practice.

## 7. Conclusions

This review identified 14 studies investigating feedback from WIST during work-related tasks. All studies used tri-axial accelerometers, with three studies using tri-axial IMUs to provide feedback on posture or movement behaviour during work-related tasks. Visual and/or vibrotactile feedback were the most common feedback strategies, with only three studies evaluating comfort and/or wearability. A low level of evidence from the 14 studies supported upper body posture and/or movement behaviour improvements using WIST feedback, but no improvements in pain. Few studies reported enough technological detail for methodological reproducibility. Thus, a minimum reporting Technology and Design Checklist for WIST studies has been proposed in this review. Moreover, higher-quality studies are needed to translate WIST systems into current ergonomic or rehabilitation practices for individuals with work-related posture or movement problems.

## 8. Key Findings

This review investigated wearable inertial sensor technology to measure upper body posture and movement behaviour and provide feedback during work or work-related activities.

Based on the low quality of studies, there was limited evidence to support the use of wearable inertial sensor feedback to change neck, upper and/or lower trunk posture, very limited evidence supporting changes in movement behaviour and limited evidence that WIST feedback improves neck and lower back pain/discomfort.

Despite the importance of user’s acceptance of technology for implementation in the workplace, wearability and/or comfort assessments were only conducted in three included studies.

Most studies lacked technological detail for methodological reproducibility; therefore, a ‘Technology and Design Checklist’ was proposed to recommend a minimum reporting standard for the technical and design methodologies of future wearable inertial sensor studies.

## Figures and Tables

**Table 1:** Search terms and strings used in the scoping review.

acceleromet* or “ambulatory monitoring” or gyroscope* or magnetomet* or “inertial sensor*” or “inertial measurement unit*” AND posture or “upper body” or workstation* or “work station*” or workplace or “occupational health” or “skeletal muscle” or “upper extremity” or arm or “upper limb*” or cervical or thoracic or spine or neck or back or shoulder* or “musculoskeletal disease*” or monitoring or msd AND wearable systems or “biomechanical phenomena” or “biomechanical feedback” or “feedback device” or movement or locomotion or “real time” or “realtime” or wireless or “chronic pain” or “reproducibility of results” or reliability or validity or “therapeutic effect” or “on-body sensor” or “Feedback effect”

**Table 2:** Summary of study characteristics for included studies.

Study	Setting	Study Population and Eligibility	Design	Objective	Comparison Groups
Brakenridge, Fjeldsoe [67]	Office	153 desk-based office workers (53 males, 34 female) Mean age (SD): 38.9, (8.0) Eligibility: ambulatory for 10 m	Cluster-randomised trial	Evaluation of organisational-support strategies compared to feedback from WIST and support to reduce sitting in office workers. Duration: 12 months	Randomised: group 1 (*n* = 87), ORG: organisational-support intervention group 2 (*n* = 66), ORG + tracker. No control group
Ribeiro, Sole [68]	Office	62 healthcare and administration workers (5 male, 57 female) Mean age (SD): 49.6, (12.4) Eligibility: with or without lower back pain.	Randomised control trial	Effectiveness of a feedback device for modifying lumbopelvic posture postural behaviour during daily work-related activities: Duration: six weeks (weeks 1–6); intervention: four weeks (weeks 2–5)	Randomised into 3 groups: constant feedback (*n* = 19); intermediate feedback (*n* = 25); or control (no feedback) (*n* = 18). Comparison between baseline (one week) and follow up (week 4). Intervention conducted for four weeks (weeks 2–5)
Thanathornwong and Suebnukarn [69]	Dental clinic	16 dental students (8 female, 8 male) Age range 21–23. Mean, SD: NR Eligibility: healthy. Health and work questionnaire	Randomised crossover 2 × 2 trial (pre-post-test)	Differences in upper trunk posture using WIST feedback during a dental procedure. Duration: NR	Same group: group A (*n* = 8) feedback; group B (*n* = 8) no feedback
Thanathornwong, Suebnukarn [70]	Student periodontal clinic	16 dental students (2 males, 14 female) Age range 21–23. Mean, SD: NR Eligibility: healthy. Health and work questionnaire	Randomised crossover 2 × 2 trial (pre-post-test)	Differences in upper trunk and neck posture using WIST feedback during a dental procedure. Duration: NR	Same group: group A (*n* = 8) feedback; group B (*n* = 8) no feedback
Vignais, Miezal [71]	Simulated industrial environment	12 male student participants Mean age (SD): 22.5, (2.5) Eligibility: Health not reported	Cross-sectional	Differences in upper body posture using WIST feedback during an industrial manual task. Duration: NR	Two groups (randomised): WR group (feedback) (*n* = 6); WOR group (control no feedback) (*n* = 6)
Ailneni, Syamala [72]	Laboratory based	19 participants (9 males, 10 females) Mean (SD): 24.47 (5.32) Eligibility: Healthy	Cross-sectional	Comparison of head and neck posture with and without feedback from WIST during computer users. Duration: 2 h	Same group: 2 × 30 min typing tasks (30 min sitting, 30 min standing) with feedback; repeated without feedback
Boocock, Naudé [73]	Laboratory based	36 university students Gender: NR Mean (SD) age: feedback group: 25.7 (4.6); no feedback 25.6 (5.1) Eligibility: healthy	Cross-sectional	Modifying lumbosacral posture in response to real-time external biofeedback during a repetitive lifting task compared to no feedback Duration: 20 min	Randomised: two groups: feedback (*n* = 18), no feedback (*n* = 18)
Bootsman, Markopoulos [74]	Hospital	13 female nurses (day shift) Mean age (SD): 39.77 (13.6) Eligibility: healthy. No lower back pain and not sedentary during work	Cross-sectional	Investigating whether feedback from WIST influences postural behaviour positively compared to no feedback. Comparison between two feedback strategies in working nurses. Duration: 3.5 h	Same group: a continuous four-phased condition
Breen, Nisar [75]	Laboratory-based	Six asymptomatic regular computer users Mean age (SD): NR Gender: NR Eligibility: healthy. No history of neck or back pain	Cross-sectional	Modifying neck postures in regular computer users with and without feedback from WIST. Duration: NR	Same group: two five-hour sessions with and without feedback during a desktop computer task (within-subject sample)
Kuo, Wang [76]	Laboratory-based	21 university students (8 male, 18 female) Mean age (SD): 23.8, (3.5) Eligibility: nonspecific neck pain	Cross-sectional	Modifying spinal postures and perceived pain severity using feedback compared to no feedback during computer use. Duration: two hours	Same group: 2 × 1 h typing task (1 with feedback; 1 h without feedback)
Park, Hetzler [77]	Sedentary work environment	31 lower back pain (13 male, 18 female) Mean age (SD): 33.1, (13.3) Eligibility: pre-existing lower back pain	Cross-sectional	Effects of postural training with vibrational biofeedback on pre-existing lower back pain during daily work-related activities. Duration: 21 days (device worn during working hours only)	Allocated into two groups: feedback (*n* = 16), no feedback (*n* = 15)
Cerqueira, Da Silva [78]	Simulated workplace environment	5 individuals (1 female and 4 males) Mean age (SD): 24.0, (1.1) Eligibility: none specified	Cross-sectional (proof of concept)	Effects of posture behaviour using biofeedback and without feedback during simulated workplace tasks. Duration: approximately 6.5 min	Same group: five continuous tasks repeated 4 times (2 times with feedback remaining 2 times without feedback)
Lind, Diaz-Olivares [79]	Simulated workplace environment	16 university staff and/or students (9 female, 9 male) Mean age (SD): 25, (8.0) Eligibility: mail sorting experience and no musculoskeletal discomfort	Cross-sectional	Effects of arm posture and movement modification using feedback during simulated mail sorting tasks. Duration: <15 min	Same group: using two experimental conditions A and B. Sorting mail with verbal ergonomic instructions or verbal instructions in combination with feedback Organising mail trays with verbal ergonomic instructions or verbal instructions in combination with feedback
Doss, Robathan [80]	Patient-handling tasks	10 nursing students (all female) Mean age (SD): 26.1 (9.1) Eligibility: no history of back pain	Cross-sectional	To provide a feedback intervention that could be implemented in a student curriculum to educate student trainees. Duration: NR	Same group: to preform three patient-handling tasks with and without feedback

NR: not reported.

**Table 5:** Wearable inertial sensor technology (WIST) system characteristics used in each of the included studies.

Study	Sensor Model	Sensor Location and Attachment	Sensor Quantity/Sampling Frequency	Filter Type/Frequency Cut-Off	Sensor Connection	Technology Readiness	Sensor Validation or Accuracy	Wearability Assessment	Reported WIST Limitations
Brakenridge, Fjeldsoe [67]	Accel * LUMOback Bodytech. ActivPal3 Pal Technologies (monitor only)	Posterior-worn sensor at the waistline	1 NR	NR	Integrated Bluetooth* sync to mobile phone	CA	MV	NR	Low uptake and self-directed usage of WIST may limit effectiveness. N = 14 (32.6%) reported using WIST device: irritation or rash (*n* = 3), uncomfortable (*n* = 8), minor back pain/strain (*n* = 3)
Ribeiro, Sole [68]	Accel Movement Metrics Ltd.	Participant’s belt (lateral position)	1 NR	NR	Integrated within device	CA	Prior validation; accuracy to 1°	NR	No time stamp of on/off periods. Error of 8° between days and 5° within days. Clothing may alter postural-pattern estimates.
Thanathornwong and Suebnukarn [69]	Accel ADXL345	Placed posteriorly onto the upper body of a gown	1 Only range 12.5–400 Hz	NR	Cable connected (sensor to computation device)	C	NR; stated accuracy of 0.01°	NR	Custom-developed software may not be effectively applied to all patients
Thanathornwong, Suebnukarn [70]	Accel ADXL345 Analog devices USA	Face shield sensor + Sensor on posterior of gown of upper body	2 Range 12.5–400 Hz	NR	Cable connected (sensor to computation device)	C	NR; Stated accuracy of 0.01°	NR	NR
Vignais, Miezal [71]	IMU (Accel, Gyros and Magne). Bi-axial goni Colibri IMU SG65 (monitor only)	Attached by an elastic strap: bilateral forearm, upper arm, head, chest, sacrum. Wrist angle measured by goniometers.	7 IMU 2 goni 100 Hz	Kalman filters (cut-off NR)	Cable connected	C	NR	NR	Inferred computations using the RULA tool. IMU errors influenced by magnetic disturbances
Ailneni, Syamala [72]	Accel Alex, NAMU inc	Posterior neck above C7 vertebra	1 NR	NR	Wireless Bluetooth	CA	NR	NR	No direct validation conducted may result in lower sensitivity in primary outcome estimates
Boocock, Naudé [73]	IMU (Accel, Gyros and Magne) * Shimmer	L1 lumbar Spinous process and sacral body. Direct to body. Attachment method: NR	2 NR	NR	wireless	CA	MV	NR	Sensor placement may interfere with other working positions
Bootsman, Markopoulos [74]	IMU (Accel, Gyros and Magne) * LSM9DSO	Sewn into a tight-fitting shirt (garment) placed over the L1 and L5 lumber vertebrae	2 NR	NR	Wireless Bluetooth	C	NR	Yes	One-size garment may not suit individual anthropometric measurements
Breen, Nisar [75]	Accel NR	C7 vertebrae sensor. Direct to body. Unable to determine mechanism for sensor attachment	1 40 Hz	NR; Low pass filtered at 10 Hz	Cable connected	C	NR	NR	Sensor measurement in single plane (sagittal)
Kuo, Wang [76]	Accel Lumo lift (Lumo Bodytech)	Taped below the left mid clavicle	1 NR	NR	Wireless	CA	MV	NR	NR
Park, Hetzler [77]	Accel Lumo lift (Lumo Bodytech)	Clip onto an undershirt 2.54 cm below the left clavicle	1 NR	NR	Wireless	CA	MV	NR	Wireless connectivity issues. Reliability and validity not evaluated prior to study. Inconsistent tracking from non-compliance during the working day
Cerqueira, Da Silva [78]	IMU (Accel, Gyros and Magne) (Invensense, USA) MPU-9250	T4 level, posterior of head and bilaterally on each upper arm. Vibration (haptic) motors: bilateral upper arms, cervical and lumbar region	4 IMUs 100 Hz 4 Haptic motors 200 Hz (vibration)	Kalman filter (cut-off NR)	Wireless	C	Validated using the UR3 robot arm. Error in full angle range 1.43% to 2.5%	Yes	NR
Lind, Diaz-Olivares [79]	IMU (Accel, Gyros and Magne) (LP Research) LPMS-B2	Velco strapped bilaterally on upper arms over a short-sleeved shirt. Vibration (haptic) motor on right upper arm	2 IMUs 25 Hz 1 vibration motor (haptic)	Kalman filter (cut-off NR)	Wireless Bluetooth	C	NR	Yes	Validation procedure and IMU drift. Potential loss of data from wireless disconnection
Doss, Robathan [80]	Accel Shimmer	Custom belt and vest	2 28 Hz	NR	Wireless Bluetooth	C	MV. Accelerometers used simultaneous with a 3D motion capture system	No	NR

NR: not reported; N/A: not applicable; MV: manufacturer validation * Information obtained from manufacturer. Technological readiness based on commercial availability: (C: custom; CA: commercially available). Accel: accelerometer; Gyro: gyroscope; Magne: magnetometer; Goni: goniometer.

**Table 6:** Risk-of-bias evaluation for randomised controlled trials (*n* = 2) using the National Institutes of Health risk-of-bias tool for controlled intervention studies.

Study	1. Study Description, Randomised RCT	2. Adequate Method of Randomisation	3. Was Treatment Allocation Concealed?	4. Providers and Participants Blinded	5. Assessors Blinded to the Participants	6. Baseline Characteristics That Could Affect Outcomes	7. Dropout Rate at an Endpoint of 20% or Lower	8. Dropout Rate at an Endpoint of 15% or Lower	9. High Adherence to Intervention Protocols in Each Group	10. Other Interventions Avoided or Similar in the Group	11. Outcomes Assessed Using Valid and Reliable Measures	12. Sample Size Sufficient to Detect Differences in Outcome	13. Outcomes Reported or Subgroups Analysed	14. Randomised Participants Analysed in Original Group	Quality Rating
Brakenridge, Fjeldsoe [67]	+	+	-	-	-	-	-	-	NR	NR	CD	+	-	+	Fair
Ribeiro, Sole [68]	+	+	+	+	+	NR	NR	NR	NR	NR	+	+	NR	NR	Fair

Note: Abbreviations: + met criteria; - did not meet criteria (other: CD, cannot determine; NA, not applicable; NR, not reported).

**Table 7:** Risk-of-bias evaluation for pre-post study designs (*n* = 2) using the National Institutes of Health risk-of-bias tool for before–after (pre-post) studies with no control group.

Study	1. Study Question or Objective Clearly Stated	2. Eligibility/Selection Criteria	3. Participants Representative of the General/Clinical Population Concealed	4. All Eligible Participants Enrolled	5. Sample Size Large Enough	6. Intervention/Test Clearly Described	7. Valid, Reliable Clearly Defined Outcome Measures	8. Researchers Blinded to Participants’ Interventions/Exposures	9. Loss to Follow Up <20%	10. Statistical Tests of Outcomes Measured Pre-Post	11. Outcomes and Measures Conducted Multiple Times before and after Tests	12. Intervention at Group Level, Use of Individual Data at a Group Level	Quality Rating
Thanathornwong and Suebnukarn [69]	+	-	-	NR	+	+	-	-	+	Y	-	+	Poor
Thanathornwong, Suebnukarn [70]	+	-	-	NR	+	+	-	-	+	Y	-	+	Poor

NOTE: Abbreviations: + met criteria; - did not meet criteria (other: CD, cannot determine; NA, not applicable; NR, not reported).

**Table 8:** Risk-of-bias evaluation for cross-sectional studies (*n* = 7) using the National Institutes of Health risk-of-bias tool for observational cohort and cross-sectional studies.

Study	1. Research Question or Objective Clearly Stated	2. Was the Study Population Clearly Specified and Defined	3. Participation Rate of Eligible Persons ≥50%	4. Subjects Recruited from Same or Similar Populations	5. Sample Size Justification	6. Exposure(S) of Interest Measured Prior to the Outcome(S)	7. Sufficient Timeframe	8. Different Levels of the Exposure as Related to the Outcome	9. Exposure Measure Clearly Defined, Valid and Reliable	10. Exposures(S) Assessed More than Once Over Time	11. Outcomes and Measures Clearly Defined, Valid and Reliable	12. Outcome Assessors Blinded to the Exposure	13. Follow-Up after Baseline ≤20	14. Adjusted for Potential Confounding Variables	Quality Rating
Ailneni, Syamala [72]	+	-	+	NR	NR	-	-	NA	+	+	+	-	+	NA	Fair
Boocock, Naudé [73]	+	-	+	+	+	-	-	NA	+	-	+	-	+	NA	Fair
Bootsman, Markopoulos [74]	+	-	+	+	NR	-	-	NA	-	+	-	-	+	NA	Fair
Breen, Nisar [75]	-	-	+	NR	-	-	-	NA	-	+	-	-	NA	NA	Poor
Kuo, Wang [76]	+	-	+	+	-	-	-	NA	+	+	+	-	+	NA	Poor
Park, Hetzler [77]	+	-	+	+	-	-	-	NA	-	-	-	-	+	NA	Poor
Vignais, Miezal [71]	+	-	+	NR	-	-	-	NA	-	-	-	-	NA	NA	Poor
Cerqueira, Da Silva [78]	+	-	+	-	-	-	+	NA	+	+	+	-	+	NA	Fair
Lind, Diaz-Olivares [79]	+	-	+	+	-	-	+	NA	+	+	+	-	+	NA	Fair
Doss, Robathan [80]	+	-	+	+	-	-	+	NA	+	+	+	-	+	NA	Fair

NOTE: Abbreviations: + met criteria; - did not meet criteria (other: CD, cannot determine; NA, not applicable; NR, not reported).

**Table 9:** Evidence for changes in posture and movement behaviour during work or performing work-related activities.

Study	Risk-of-Bias Quality Rating	Outcome	Level of Evidence
Ailneni, Syamala [72]	Fair	Improved neck and upper and/or lower trunk posture: Sagittal plane (flexion/extension)	Limited
Breen, Nisar [75]	Poor		
Kuo, Wang [76]	Poor		
Vignais, Miezal [71]	Poor		
Thanathornwong, Suebnukarn [70]	Poor		
Thanathornwong and Suebnukarn [69]	Poor		
Ribeiro, Sole [68]	Fair		
Bootsman, Markopoulos [74]	Fair		
Boocock, Naudé [73]	Fair		
Doss, Robathan [80]	Fair		
Cerqueira, Da Silva [78]	Fair	Improved neck and upper and/or lower trunk posture: Sagittal and coronal plane (flexion/extension and lateral flexion)	
Park, Hetzler [77]	Poor	No neck and/or lower back pain/discomfort improvements	Limited
Kuo, Wang [76]	Poor		
Brakenridge, Fjeldsoe [67]	Fair	Improved movement behaviour (Increased work stepping time)	Very limited
Cerqueira, Da Silva [78]	Fair	Reduced upper-arm elevation angle or accumulative time	Limited
Lind, Diaz-Olivares [79]	Fair		

**Table 10:** Technology and Design Checklist.

Data Collection Inertial Sensor	WIST Processing/Analysis	Feedback Parameters	Study Design
Sensor ▪ Model/manufacture ▪ Inertial sensor type ▪ Quantity ▪ Connection method ▪ Anatomical location ▪ Attachment method	▪ Frequency sampling rate ▪ Filter type (e.g., Butterworth) and cut-off frequency ▪ Fusion type (e.g., Kalman) ▪ Processing system ▪ 3D joint/modelling angle(s)/rotation(s) * ▪ Joint coordinate system ▪ Algorithm origin/availability	▪ Trigger (kinematic set-point) ▪ Biomechanical set-point source/origin ▪ Content ‡ ▪ Timing (latency) † ▪ Frequencies of feedback occurrences ▪ Monitoring duration (h, min) ▪ Source/device of feedback ▪ Participant evaluation on feedback content/timing ▪ Suggested technology readiness for clinical application ▪ Limitations	▪ Refer to STROBE statement checklists [137] ▪ Prior assessment of WIST validity/reliably with outcomes reported ▪ Follow-up evaluation

* Refer to International Society of Biomechanics (ISB): https://isbweb.org/ (accessed on 2 February 2021) or if not using standardised methods, the provision of equivalent information to replicate is required. ‡ Visual, audible, vibrotactile, multimodal, other; † concurrent, terminal, fading, other.

## Data Availability

Not applicable. Database searches were conducted only for this scoping review.
